# Genomic diversity in functionally relevant genes modifies neurodevelopmental versus neoplastic risks in individuals with germline PTEN variants

**DOI:** 10.21203/rs.3.rs-3734368/v1

**Published:** 2023-12-14

**Authors:** Charis Eng, Adriel Kim, Lamis Yehia

**Affiliations:** Cleveland Clinic

## Abstract

Individuals with germline *PTEN* variants (PHTS) have increased risks of the seemingly disparate phenotypes of cancer and neurodevelopmental disorders (NDD), including autism spectrum disorder (ASD). Etiology of the phenotypic variability remains elusive. Here, we hypothesized that decreased genomic diversity, manifested by increased homozygosity, may be one etiology. Comprehensive analyses of 376 PHTS patients of European ancestry revealed significant enrichment of homozygous common variants in genes involved in inflammatory processes in the PHTS-NDD group and in genes involved in differentiation and chromatin structure regulation in the PHTS-ASD group. Pathway analysis revealed pathways germane to NDD/ASD, including neuroinflammation and synaptogenesis. Collapsing analysis of the homozygous variants identified suggestive modifier NDD/ASD genes. In contrast, we found enrichment of homozygous ultra-rare variants in genes modulating cell death in the PHTS-cancer group. Finally, homozygosity burden as a predictor of ASD versus cancer outcomes in our validated prediction model for NDD/ASD performed favorably.

## INTRODUCTION

*PTEN* hamartoma tumor syndrome (PHTS) is a molecular diagnosis encompassing all individuals who have pathogenic/likely pathogenic germline *PTEN* variants, irrespective of phenotype.^1,2^ PTEN, a phosphatase and tensin homolog, is a tumor suppressor that has diverse functions, including regulating cellular growth, proliferation, and apoptosis, as well as DNA repair and maintenance of genomic stability.^2–6^ It is, therefore, not surprising that germline *PTEN* alterations can give rise to a wide spectrum of clinical features associated with overgrowth, most notably macrocephaly and hamartomatous lesions involving various tissues, and higher lifetime risks of developing breast, thyroid, and other cancers.^7–13^ Intriguingly, up to 17% of individuals with autism spectrum disorder (ASD) with macrocephaly are found to carry germline *PTEN* variants, and recent case studies suggest that up to 50% of children with *PTEN* variants are diagnosed with ASD.^14–18^

*PTEN* is now considered one of the most common monogenic ASD-predisposition genes.^19,20^ Phenotypic trait variations, whether considered polymorphic or disease-related, could fundamentally be the result of individual genomic diversity, which often is measured as heterozygosity reflecting the distinct combination of alleles carried in a given individual.^21^ Human genomic diversity has been investigated to demonstrate the advantage of higher heterozygosity, hence, diversity, in fitness-related traits, and more recently, in association with improved response to and survival with immunotherapy in cancer patients.^22–24^ Many studies have shown that germline human leukocyte antigen (HLA) class I diversity serves as a strong determinant of survival of cancer patients after receiving immune checkpoint inhibitor treatment.^23–27^ Although these studies focused on narrowly defined regions of the genome, namely major histocompatibility complex (MHC) class I, in cancer patients and their responses to treatment, this highlights the intriguing hypothesis that differences in germline genomic diversity may predispose to the development of a particular phenotype amongst those with identical monogenic etiology and may serve as an intrinsic marker of disease or phenotype development at the individual level.

Little known until recently, PHTS manifests with a range of immune-related phenotypes, including autoimmunity, lymphoproliferation, and immunodeficiency, adding to the continuously expanding complexity of PTEN biology.^28–30^ A recent study showed that PHTS individuals’ class II HLA genotypes may modulate autoimmunity and immune dysregulation by cross-talking with the gut microbiome.^31^ However, what remains elusive is whether the global genomic background, versus differences limited to the HLA loci, could be associated with phenotypic differences in PHTS, particularly as related to disparate clinical phenotypes such as cancer and ASD. Thus, we sought to analyze differences in germline genomic diversity as a modifier of neurodevelopmental phenotypes, including ASD, versus non-neurodevelopmental phenotypes, such as cancer, in individuals with PHTS.

## RESULTS

### Research participants and study design

**Figure 1:**
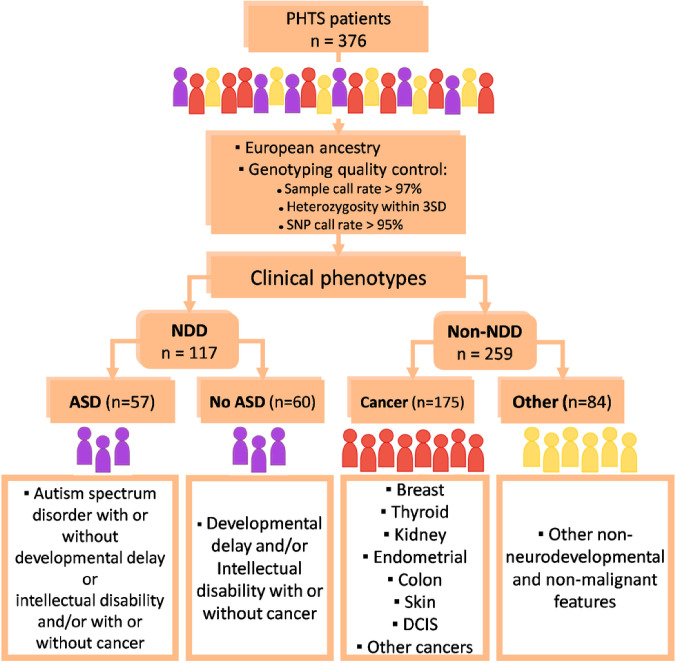
Overview of study design and study participants. Individuals of European ancestry with PTEN hamartoma tumor syndrome (n=376) were genotyped and grouped according to the clinical phenotypes of interest.

**Figure 2:**
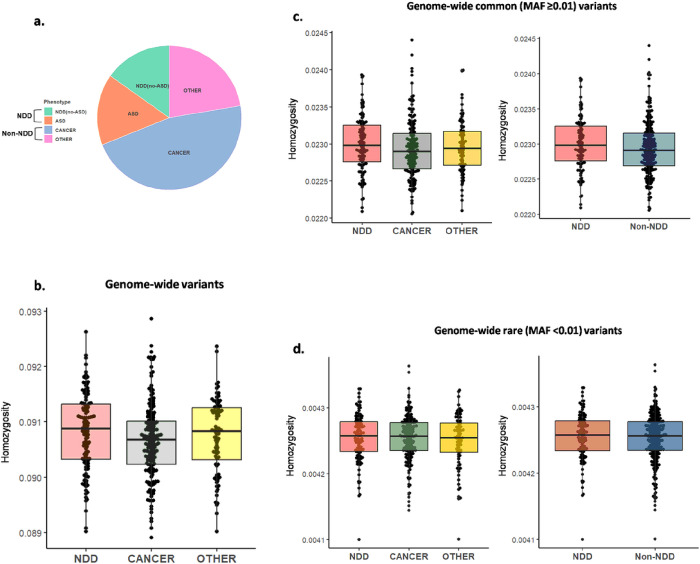
Non-significant increase of genome-wide homozygosity in PHTS-NDD is observed. **a** The homozygosity burden of genome-wide homozygous variants, which include all detected alternate alleles, are non-significantly increased in PHTS-NDD (one-way ANOVA *p*=0.142), and especially when compared to PHTS-cancer (two-tailed t-test *p*=0.053). **b**The increased homozygosity observed with genome-wide all variants are consistent in the common variants (MAF ≥0.01) in PHTS-NDD compared to PHTS-cancer (two-tailed Mann-Whitney test *p*=0.07), PHTS-other (two-tailed t-test *p*=0.5) and non-NDD (two-tailed Mann-Whitney test *p*=0.1), which combines PHTS-cancer and PHTS-other patients. **c** The homozygosity burden with genome-wide rare variants (MAF <0.01) show no conspicuous differences between PHTS groups (PHTS-NDD vs. PHTS-cancer, two-tailed Mann-Whitney test *p*=0.8; PHTS-NDD vs. PHTS-other, two-tailed Mann-Whitney test *p*=0.7; PHTS-NDD vs. PHTS non-NDD, two-tailed Mann-Whitney test *p*=0.7). **d**The pie chart shows the relative distribution of PHTS study groups according to the clinical phenotype.

A total of 376 PHTS patients of European ancestry whose DNA passed genotyping quality control were analyzed in this study. These patients were parsed into two broad phenotypic groups in the first instance. PHTS-NDD, from which PHTS-ASD was sub-grouped, and PHTS non-NDD, from which PHTS-cancer and PHTS-other were sub-grouped (Fig. 1). In the second instance, we considered patients in the PHTS-cancer and PHTS-other groups collectively as PHTS non-NDD, as it cannot be ascertained whether patients in the PHTS-other group will develop cancer over time while we are confident that the patients in the PHTS-other group do not have ASD, DD, or ID (Fig. 2a). Individuals in the PHTS-NDD group had a younger age at consent (median [IQR], 8 [4–18] years old) compared to PHTS-cancer (median [IQR], 50 [43–60] years old) and PHTS-other (median [IQR], 34 [16–45] years old) (Table 1). Male sex was overrepresented in the PHTS-NDD group (69%), while the female sex was overrepresented in the PHTS-cancer group (82%). There were no differences in the types of germline *PTEN* variants found in the study participants across the phenotypic groups. The highest percent of *PTEN* variants that were pathogenic/likely pathogenic is 92% in PHTS-NDD, 75% in PHTS-cancer, and 87% in PHTS-other. While variation has not been shown to be associated with high- and moderate-penetrance disease predisposition, common variants have been shown to act as modifiers. Thus, we examined genome-wide homozygosity enrichment of common variants (MAF ≥ 0.01), rare variants (MAF < 0.01), and ultra-rare variants (MAF < 0.0001).

### Homozygosity of common variants in PHTS-NDD/ASD is enriched in genomic regions crucial to human development

All patients were genotyped using the same SNP-array. The variants were defined as all non-reference alleles detected in the study series and subjected to homozygosity calculation. We first investigated genome-wide homozygosity. We observed a non-significantly increased burden of genome-wide homozygous variants in PHTS-NDD (one-way ANOVA *p* = 0.142) especially when compared to PHTS-cancer (two-tailed t-test *p* = 0.053) (Fig. 2b). The non-significantly increased homozygosity was consistent in the subsequent analysis of common variants (MAF > 0.01) in PHTS-NDD versus all other phenotype groups (Fig. 2c–d).

**Figure 3:**
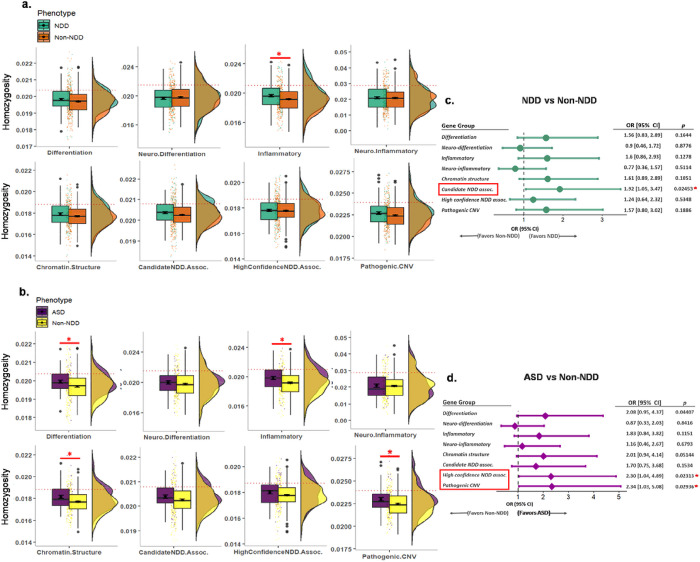
Homozygous common variants in PHTS-NDD and PHTS-ASD are significantly enriched in gene groups germane to NDD/ASD pathobiology. **a** The overall increased burden of homozygous common variants in PHTS-NDD was significantly enriched with genes involved in inflammatory processes (adjusted two-tailed unpaired t-test *p*=0.040). **b**The overall increased burden of homozygous common variants in PHTS-ASD were significantly enriched with genes involved in differentiation, inflammatory processes, and chromatin structure regulation and pathogenic CNV regions (adjusted two-tailed unpaired t-test *p*=0.040, *p*=0.040, and *p*=0.040, and adjusted two-tailed Mann-Whitney test *p*=0.045, respectively). **c**The association analysis of relatively higher burden of homozygous common variants in PHTS-NDD was found with the candidate NDD-associated genes (Fisher’s exact test *p*=0.025). **d** Relatively higher burden of homozygous common variants in PHTS-ASD were found with the high-confidence NDD-associated genes and pathogenic CNV regions (Fisher’s exact test *p*=0.023 and *p*=0.029, respectively).

To infer the biological implications of the NDD-associated increased homozygosity in common variants, we identified several groups of functionally relevant genes, including genes involved in cellular differentiation, inflammatory processes, and chromatin structure regulation. Additionally, two sets of previously curated genes known to be associated with NDD with low and high confidence were also used in the analysis and are referred to as candidate NDD-associated and high confidence NDD-associated genes, respectively.^32^ Finally, genomic regions known to carry pathogenic copy number variations (CNV) were also included in the analysis.^33,34^ The homozygosity of common variants within each of these selected genomic regions were measured per PHTS individual and compared between the NDD and non-NDD groups. Two different statistical approaches were applied to evaluate the burden of homozygosity. First, the overall burden of homozygous common variants was assessed by calculating the ratio of homozygous variants to the total number of genotyped loci in each individual. The results showed that the increased burden of homozygous common variants in PHTS-NDD was significantly enriched with genes involved in inflammatory processes (adjusted two-tailed unpaired t-test *p* = 0.040) and in PHTS-ASD, significantly enriched with genes involved in differentiation, inflammatory processes, and chromatin structure regulation (adjusted two-tailed unpaired t-test *p* = 0.040 for all comparisons) as well as pathogenic CNV regions (adjusted two-tailed Mann-Whitney test *p* = 0.045) (Fig. 3a–b). Next, the relatively high burden of homozygous common variants from each gene set with PHTS-NDD and PHTS-ASD was tested by combining the PHTS-NDD and PHTS non-NDD datasets and establishing above-1SD as a criterion of having higher burden of homozygous common variants. This approach was also applied to the PHTS-ASD and PHTS non-NDD datasets. We observed a higher burden of homozygous common variants associated with candidate NDD-associated genes in PHTS-NDD (Fisher’s exact test *p* = 0.025) and associated with high-confidence NDD-associated genes as well as pathogenic CNV regions in PHTS-ASD (Fisher’s exact test *p* = 0.023 and *p* = 0.029, respectively) (Fig. 3c–d). These findings were consistent with subset analyses of the burden of homozygous common variants in PHTS-NDD and PHTS-ASD only limited to individuals with pathogenic/likely pathogenic *PTEN* variants (**Supplementary Fig. 1**).

**Figure 4:**
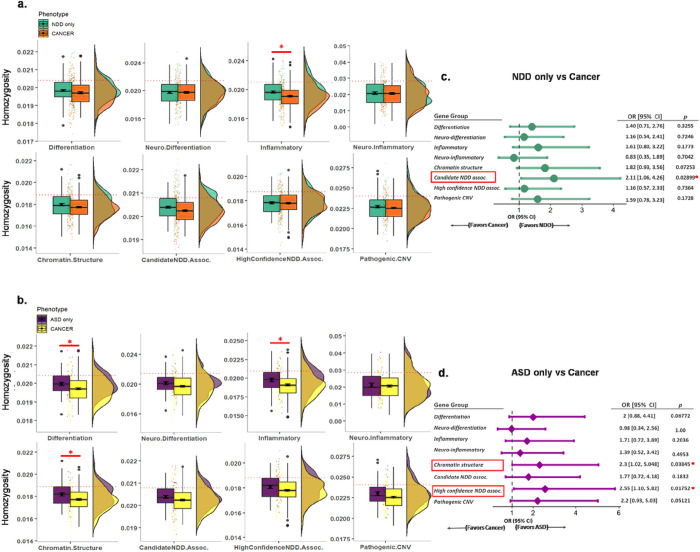
The gene groups enriched by the homozygous common variants are consistent in PHTS-NDD and PHTS-ASD excluding those co-morbid with cancer when compared to PHTS-cancer. **a** The overall burden of homozygous common variants in PHTS-NDD-only, denoting those co-morbid with cancer, was consistently enriched within genes involved in inflammatory processes (adjusted two-tailed unpaired t-test *p*=0.040). **b**The overall burden of homozygous common variants in PHTS-ASD only was consistently enriched within genes involved in differentiation, inflammatory processes, and chromatin structure regulation (adjusted two-tailed unpaired t-test *p*=0.040, *p*=0.040, and *p*=0.040, respectively). **c** The association of relative burden analysis in PHTS-NDD only against PHTS-cancer was consistent with candidate NDD-associated genes (Fisher’s exact test *p*=0.029). **d** The relative homozygous common variants burden in PHTS-ASD only against PHTS-cancer was associated with the genes of chromatin structure regulation and high-confidence NDD-associated genes (Fisher’s exact test *p*=0.038 and *p*=0.018, respectively).

Subsequent analyses to compare NDD/ASD phenotype with those currently with cancer were performed by removing the patients co-morbid with cancer from the PHTS-NDD/PHTS-ASD groups (hence, PHTS-NDD/PHTS-ASD only) and conducting the same analysis against PHTS-cancer, a sub-group of PHTS non-NDD. The overall burden of homozygous common variants was consistently enriched in the PHTS-NDD group, only within genes involved in inflammatory processes (adjusted two-tailed unpaired t-test *p* = 0.040), and in PHTS-ASD, only within genes involved in differentiation, inflammatory processes, and chromatin structure regulation (adjusted two-tailed unpaired t-test *p* = 0.040 for all comparisons) (Fig. 4a–b). While the association of relative burden analysis in PHTS-NDD-only against PHTS-cancer was consistent with candidate NDD-associated genes (Fisher’s exact test *p* = 0.029), the relative homozygous common variant burden in PHTS-ASD-only against PHTS-cancer was associated with genes related to chromatin structure regulation and high-confidence NDD-associated genes (Fisher’s exact test *p* = 0.038 and *p* = 0.018, respectively) (Fig. 4c–d).

### Pathway enrichment analysis of genes with qualifying homozygous variants reveals biological pathways and functions germane to neurodevelopmental phenotypes

**Figure 5:**
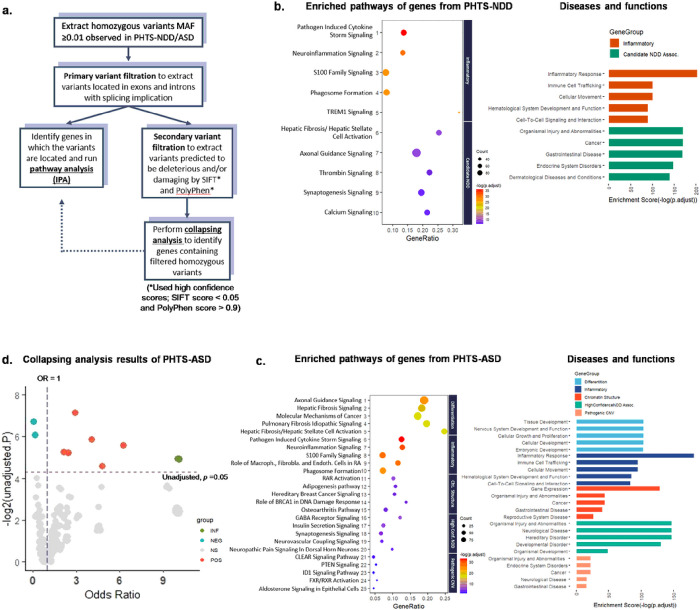
Stringent variant filtration identifies genes leading to enriched biological pathways and suggestive modifier genes for PHTS-NDD and PHTS-ASD. **a** Two-step variant filtration was performed to conduct consecutive analyses of the genes carrying the homozygous common variants that met the filtration criteria. Primary filtration was applied to identify the genes for subsequent pathway analysis followed by more stringent secondary filtration to identify the genes for collapsing analysis to infer modifier NDD/ASD genes. **b** Among the enriched pathways of the genes carrying primarily filtered variants PHTS-NDD were the biological pathways pertinent to neurobiology of NDD. **c** The enriched pathways of the genes carrying primarily filtered variants from PHTS-ASD also showed the biological pathways very pertinent to neurobiology of ASD. Moreover, the annotated diseases and functions of the pathways implicate neurobiological and developmental processes and diseases/disorders. **d** Collapsing analysis of the filtered homozygous common variants from PHTS-ASD identifies nine positively and two negatively ASD-associated genes with suggestive significance.

The homozygous common variants from the gene sets and genes within pathogenic CNV regions that were significant for enrichment in PHTS-NDD and PHTS-ASD were filtered by primary criteria and subjected to downstream pathway enrichment analysis (Fig. 5a). Collectively, the results revealed several biological pathways pertinent to NDD and ASD neurobiology. In particular, neuroinflammation, axonal guidance, and synaptogenesis signaling were the commonly enriched pathways for PHTS-NDD and PHTS-ASD (Fig. 5b–c). Additional molecular neurobiological pathways, such as GABA receptor signaling, neurovascular coupling, and neuropathic pain signaling, were detected from within the high confidence NDD-associated genes for PHTS-ASD (Fig. 5c). The annotated functions from the enriched pathways implicate tissue and system development processes, including cellular, embryonic, reproductive, and nervous system, and immune related functions, such as inflammatory response and immune cell trafficking. Moreover, the noted diseases from the enriched pathways were particularly implicated in neurological diseases and developmental disorders (Fig. 5c).

### Homozygosity of ultra-rare variants in PHTS-cancer is enriched in genes modulating cellular death

**Figure 6:**
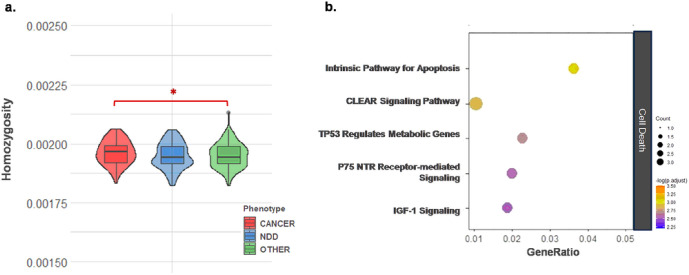
Homozygosity of ultra-rare variants in PHTS-cancer are enriched in cell death genes. **a** The overall burden of homozygous rare variants in PHTS-cancer was found to be significantly enriched with genes involved in cell death processes (Unadjusted one-way ANOVA *p*=0.044; Cancer vs. NDD, unadjusted two-tailed unpaired t-test *p*=0.021; Cancer vs. Other, unadjusted two-tailed unpaired t-test *p*=0.026). **b**Enriched pathways of the genes identified to carry the filtered homozygous ultra-rare variants in PHTS-cancer show top five canonical pathways above unadjusted *p*-value of 0.05.

In the homozygous variants analysis of functionally relevant genes in relation to the NDD phenotype, we observed a nonsignificant increase in the burden of homozygous *rare* variants in chromatin structure regulating genes in the cancer group (**Supplementary Fig. 2**). This finding led us to speculate whether the burden of rare variants in terms of homozygosity might be different in the PHTS-cancer group and prompted us to further investigate rare variants in the biological context relating to cancer etiology. We identified several groups of functionally relevant genes that play a role in the cell cycle, cell death, DNA damage, oncogenesis, and tumor suppression. The homozygosity of common and rare variants within each of these gene groups were measured and compared across cancer, NDD, and other (representing non-malignant and non-neurodevelopmental) groups. The comparative analysis of both common and rare variants showed no statistically significant differences across the PHTS groups but suggested an increase in homozygous rare variants in the cancer group (**Supplementary Fig. 3**). We then subsetted out the ultra-rare variants with MAF < 0.0001 from the rare variants and performed the burden testing for further comparison. We found significant enrichment of homozygous ultra-rare variants in genes modulating cell death in the cancer group (Fig. 6a). The homozygous ultra-rare variants found in the cancer group were filtered to extract the ones located in exons and introns with splicing implication and subjected to pathway enrichment analysis. The results revealed biological pathways known to be involved in tumorigenesis, such as intrinsic pathway for apoptosis, CLEAR signaling, and TP53 regulated metabolic pathway (Fig. 6b).

### Collapsing analysis of qualifying homozygous variants suggests candidate NDD and ASD modifier genes in PHTS

By applying more stringent secondary filtering criteria for the homozygous variants detected in our research participants, we then sought to infer candidate modifier genes for the NDD and ASD phenotypes in PHTS (Fig. 5a). The non-weighted burden approach of collapsing homozygous variants analysis was used to identify genes carrying the qualifying homozygous variants in PHTS-NDD and PHTS-ASD compared with PHTS non-NDD (Fig. 5d and **Supplemental Fig. 4**). The analysis resulted in 12 suggestive candidate NDD modifier genes and 11 suggestive candidate ASD modifier genes, many of which were implicated in the pathway enrichment analysis (above). Among the suggestive candidate NDD modifier genes, *GABRA4* (OR = 2.07, unadjusted *p* = 0.029), *OPRM1* (OR = infinite, unadjusted p = 0.030), *LRP8* (OR = 0.47, unadjusted p = 0.031), *LAMA5* (OR = 9.08, unadjusted p = 0.034), and *TLR3* (OR = 0.28, unadjusted *p* = 0.042), are particularly implicated in pathways pertinent to neuropathology, such as neuroinflammation, CREB signaling, synaptogenesis, and myelination (Table 2). *TBTBD13, LRP8* and *TLR3* were negatively associated with the NDD phenotype. Similarly, several suggestive candidate ASD modifier genes, including *LRP8* (OR = 0.25, unadjusted *p* = 0.015), *CX3CR1* (OR = 4.21, unadjusted *p* = 0.016), *BST1* (OR = infinite, unadjusted *p* = 0.032), and OPRM1 (OR = infinite, unadjusted *p* = 0.032) are implicated in neuropathology, including inflammation, synaptogenesis, and CREB signaling in neurons (Table 3). Notably, 3 out of the 11 ASD genes are listed as SFARI (Simons Foundation Autism Research Initiative) genes with respective scores of 2, indicating a strong association with ASD. Additionally, another 4 out of the 11 ASD modifier genes are not reported SFARI genes but have at least one gene from the same gene family with SFARI scores ranging from 1 to 3 (score of 1 being high confidence, 2 being strong evidence, and 3 being suggestive evidence).

### Internally validated prediction models demonstrate the feasibility of employing homozygosity burdens as predictors of NDD and ASD phenotypes in PHTS

**Figure 7:**
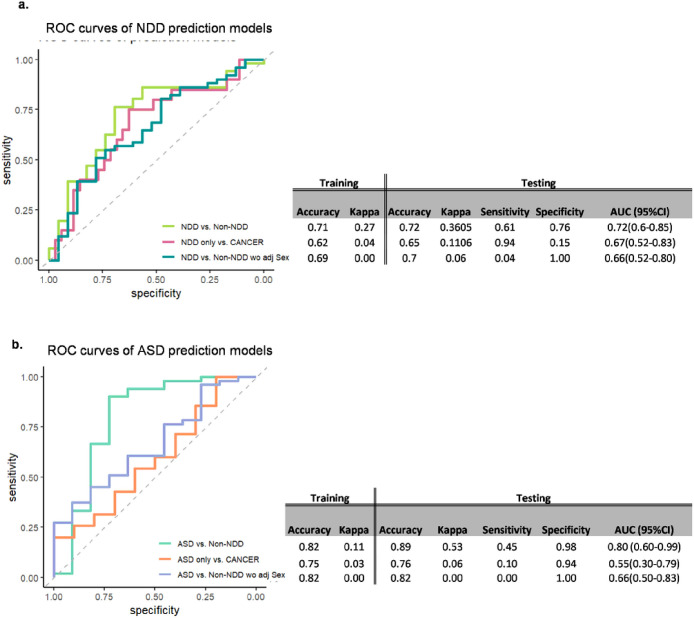
Internally validated prediction models demonstrate the feasibility of employing homozygosity burdens as predictors for NDD and ASD phenotypes in PHTS. **a** Receiver operating characteristic curves predicting NDD vs. non-NDD and NDD only vs. cancer. Note that NDD only vs. cancer model is without adjusting for sex. The model for NDD vs. non-NDD without adjusting for sex is depicted to demonstrate the model performance of NDD vs. non-NDD using the selected homozygosity burden predictors only. **b**Receiver operating characteristic curves predicting ASD vs. non-NDD and ASD only vs. cancer. The model for ASD vs. non-NDD without adjusting for sex is depicted to demonstrate the model performance of ASD vs. non-ASD using the selected homozygosity burden predictors only.

Being able to predict whether a certain individual patient will develop NDD/ASD or cancer is a crucial goal to achieve more precise clinical management of a person with PHTS at the individual level and at the earliest ages for ASD. Hence, homozygosity burden measured in the gene sets and regions from the previous targeted analyses were used as predictors in the building of prediction models for NDD and ASD phenotypes in PHTS. The optimal predictors were selected by the backward stepwise selection procedure with initial models saturated with and regressed on homozygosity burdens calculated in each gene set and the regions from the targeted analyses and used in building the final models. Four final models were built to evaluate predictability of NDD and ASD phenotypes from non-NDD after adjusting for sex of the study participants, and NDD without cancer and ASD without cancer (NDD only and ASD only, respectively) from cancer phenotype in PHTS without adjusting for sex (**Supplementary Table 1**) The models of NDD only/ASD only versus cancer were not adjusted for sex considering the male predominance in PHTS-NDD/ASD and the female predominance in PHTS-cancer due to the high prevalence of patients with breast cancer in PHTS.^13^ The resulting performance of NDD vs. non-NDD model presented with 72% accuracy and 72% area under the curve (AUC), while the NDD only vs. cancer model presented with 65% accuracy and 67% AUC (Fig. 7a). Likewise, the classification performance of the ASD vs. non-NDD model presented with 89% accuracy and 80% AUC, while the ASD only vs. cancer model presented with 76% accuracy and 55% AUC (Fig. 7b).

## DISCUSSION

In this study, we observed that an increased genome-wide burden of homozygous variants in PHTS patients with neurodevelopmental phenotypes compared to those with non-neurodevelopmental phenotypes, specifically cancer. This increased burden of homozygous variants in NDD is found to be comprised of common variants (MAF ≥ 0.01). In contrast, the increased burden of homozygous ultra-rare variants (MAF < 0.0001) was found in PHTS-cancer compared to PHTS-NDD. Importantly, the NDD (including ASD)-associated common variants were significantly enriched in several groups of functionally related and relevant genes belonging to biological processes known to be important in neurodevelopment, and previously validated genes and genomic regions curated for neurodevelopmental disorders, including ASD. The study suggests reduced genomic diversity, thus increased homozygosity, in PHTS patients with neurodevelopmental phenotypes.

Our observations suggest that PHTS patients with PHTS-NDD carry cumulatively increased homozygous variants in inflammatory genes. Individuals with PHTS-ASD, a more specific neurodevelopmental phenotype, carry homozygous variants in additional gene groups, including those associated with differentiation and chromatin structure regulation. Consistent evidence supports the linkage between inflammation and increased risk of developing neurodevelopmental disorders. *Pten* mouse models have shown that Pten loss-of-function increases the susceptibility to immune dysregulation and abnormal activation of immune mediators, including microglia, and induces the neuroinflammation pathway.^35^ Additionally, prenatal exposure to maternal immune activation affects brain connectivity and other processes of fetal brain development, making the fetus vulnerable to developing neurodevelopmental phenotypes.^36–38^ Considering that the active development of the human brain is a process that starts from the embryonic phase and continues throughout childhood, abnormal and/or chronic exposure to inflammation prenatally and postnatally could pose increased risk of neurodevelopmental disorders. Thus, one could postulate that the germline variant affecting a gene, *PTEN*, that plays a proven role in neuroinflammation, and the reduced diversity in inflammation pathway cross talk to lower the neuroinflammation threshold of disease, here NDD/ASD.

The additional (beyond inflammation-related) gene groups enriched with homozygous variants in PHTS-ASD imply that the inflammatory process is a shared etiology between non-ASD NDD and ASD and suggests that more refined neurodevelopmental phenotypes like ASD are possibly driven by additional biological processes, such as differentiation and chromatin structure regulation in PHTS. Indeed, cellular differentiation is a key biological process during embryonic and postnatal development, and neuronal differentiation and function, in particular, have been repeatedly reported as convergent biological processes in several neurodevelopmental disorders, including ASD.^39–43^ During differentiation, constant turning on and off of gene expression requires efficient modification of DNA. Indeed, it is well established that differentiation and chromatin structure regulation are tightly coordinated and crucial during human development.^44–47^

Genome-wide association studies (GWAS) represent a popular approach to identify genomic association driven by common variants with common diseases and to determine the biological cause of heritable phenotypes.^48,49^ However, the identified disease-associated variants explain only a small portion of affected cases.^50,51^ Rare variant burden analysis has also been employed to fill the gap of the missing heritability dilemma posed by GWAS, but similarly explains a smaller portion of affected cases.^52,53^ Another pitfall is that both GWAS and rare variant burden analysis approaches require thousands of samples to achieve statistical power. This makes it very challenging to apply these standard approaches to rare genetic disorders, including PHTS, for which the sample size is often limited. Our genome-wide scale analysis of homozygous variants used in this study has not been a common approach to investigate germline variants especially in association with complex diseases, such as cancer or neurodevelopmental disorders. Such a repurposed approach is not only innovative but also necessary, especially to tackle the underlying genomic differences among PHTS patients who manifest with a wide spectrum of clinical features and develop two very disparate phenotypes, such as cancer and NDD/ASD.

Our data here posit an intriguing idea that the reduced genomic diversity collectively found within the groups of functionally relevant genes crucial for human neurodevelopment could function as a genomic modifier for the development of neurodevelopmental phenotypes in those with germline *PTEN* variants/PHTS. Accordingly, we found that increased accumulation of homozygous common variants, considered to have small effects, in the genes converging on biological processes important to neurodevelopmental processes are associated with the risk of developing NDD/ASD phenotype. Pathway enrichment analysis of the homozygous variants corroborates our observation (of reduced genomic diversity in NDD/ASD phenotype) by revealing several pathways, including synaptogenesis and axonal guidance signaling, which have been repeatedly reported through other studies to be associated with neurodevelopmental phenotypes.^39–42,54^ To take this argument further, the modifier genes suggested through our collapsing analysis, especially for ASD, identified several specific genes that are either themselves SFARI genes with strong evidence of association with ASD or have other genes of the same gene family reported in SFARI with strong evidence. We postulate that these overlapping findings in our PHTS patients suggest that the reduced genomic diversity in the set of specific biological processes could crosstalk with altered PTEN pathway(s) to modify the risk of developing neurodevelopmental phenotypes in PHTS.

In contrast, we observed an increased burden of homozygous ultra-rare variants in genes modulating cell death in the PHTS-cancer group. As the implications of common and rare variants in the contribution to phenotypic traits differ, our finding leads us to speculate that rare variants with larger effect size are associated with cancer development by converging on genes involved in cell survival-related biological processes, especially in the context of heritable dysfunction involving a well-known apoptosis gene/pathway, PTEN.

Finally, our study not only suggests a novel concept of differential genomic diversity as a modifier of neurodevelopmental and malignant phenotypes in those with germline *PTEN* variants, but also proposes potential clinical utility, especially for neurodevelopmental phenotypes, for better PHTS patient management in the future. The earlier we can predict those who will develop NDD/ASD and begin neurobehavioral therapy, the better the clinical outcome. As such, we have taken our results of quantified homozygosity into building and validating prediction models toward NDD/ASD phenotypes in PHTS. Such a phenotype prediction validation in PHTS demonstrates a positive outlook for the translatability of these findings, as we strive to build more comprehensive omics data for our PHTS patients. Using quantified homozygosity is only a first step in our model building. Including other modifiers such as CNV and mitochondrial DNA load and variation^34,55^ may prove synergistically utile.

## METHODS

### Research participants

Research participants were recruited under Cleveland Clinic institutional review board (IRB) protocols 8458 and 15–174. Principles followed by our IRB are aligned with the Declaration of Helsinki, the Belmont Report and the Common Rule. Peripheral blood was collected and used to extract DNA for genotyping and confirming the presence of germline *PTEN* variants (PHTS molecular diagnosis). Patients were divided into four groups according to their clinical phenotypes: neurodevelopmental disorders (NDD), autism spectrum disorder (ASD), cancer, and other (operationally defined as having neither cancer nor NDD/ASD). Patients having ASD, developmental delay (DD), and/or intellectual disability (ID) with or without cancer are encompassed within the PHTS-NDD grouping. A subset of patients in the PHTS-NDD group formally diagnosed with ASD (according to the Diagnostic and Statistical Manual of Mental Disorders guideline IV) with or without DD/ID and/or cancer are considered as PHTS-ASD. At the time of study consent, patients with a previous or current diagnosis of one or more cancers are considered as PHTS-cancer for the purposes of this study.^8^ Patients who have stage 0 breast cancer, such as ductal carcinoma in situ (DCIS), are also considered as a part of the PHTS-cancer group. As a result, while some patients in the PHTS-NDD/ASD group had cancer, none of the patients in the PHTS-cancer group had neurodevelopmental disorder features, such as ASD, DD, and/or ID. Patients without ASD, DD, ID, and cancer but with other neurological (eg, macrocephaly) and/or non-malignant (eg, lipoma, tissue overgrowth) features are assigned as PHTS-other. As it is impossible to ascertain whether patients in the PHTS-other group will develop cancer or not prospectively, but known to be without NDD/ASD, DD, and/or ID, patients from the PHTS-cancer and PHTS-other groups are collectively considered as PHTS non-NDD.

#### PTEN mutation analysis and selection criteria

Patients accrued for this study have germline *PTEN* variants with pathogenicity classifications ranging from variants of unknown significance (VUS), likely benign, benign and likely pathogenic to pathogenic. Patients with benign, likely benign, and VUS *PTEN* variants were included in the study because they display various clinical presentations of PHTS, including thyroid nodules, macrocephaly, gastrointestinal polyps, skin tags, lipomas, Hashimoto’s disease, and others, with or without NDD features and/or cancer including DCIS. For all analyses conducted, sub-analyses of patients with pathogenic and likely pathogenic *PTEN* variants were also performed separately. To be conservative, *PTEN* promoter variants were included in the study only if the variants had been previously either reported in association with PHTS or known to affect *PTEN* function.^8,56–58^ Pathogenicity of *PTEN* variants was determined based on orthogonal reports from CLIA (Clinical Laboratory Improvement Amendments) certified laboratories, ClinVar database classifications, and/or the ClinGen gene-specific criteria for *PTEN* variant curation.^59^

### Genotyping data and quality control

DNA samples from study patients were evaluated to ensure quality using spectrophotometry (NanoDrop 1000; Thermo Fisher Scientific, Waltham, MA, USA) and a double-stranded DNA high-sensitivity assay kit (Qubit; Thermo Fisher Scientific).^34^ All patients were genotyped using the Infinium Global Screening Array-24, version 1.0 (Illumina) at the Broad Institute Genomic Services (Cambridge, MA, USA). The array assay has a total of 642,824 variant markers covering autosomes, sex chromosomes, and mitochondrial DNA. Principal component analysis (PCA) was performed to stratify the population structure of the study cohort. Autosomal SNPs with genotyping rate < 98% and deviation from Hardy-Weinberg equilibrium (*P* ≤ 0.001) before pruning for linkage disequilibrium were filtered in PLINK version 1.927 using default parameters.^34^ Population stratification showed that most of our patients are of European ancestry, and only the patients identified as such by PCA were subjected for further quality control steps and analysis. For individual sample level quality control, sex chromosome markers were used to remove patient samples with sex discordance. Only the autosomal markers were subjected to further individual sample and SNP marker quality control. Individual samples with a heterozygosity rate ≥ 3 standard deviations and with a missing genotype rate > 3% (hence, sample genotyping success rate < 97%) were excluded from the study. For SNP marker level quality control, any autosomal marker with genotype missingness > 5% (hence, SNP genotyping success rate < 95%) were excluded from the downstream analysis.^60^

### Curation of gene sets for context-specific analysis

Gene sets for biological processes relevant to PHTS phenotypes were curated using the Gene Ontology database (GO; http://geneontology.org/). The GO database has more than 3,800 carefully defined phrases called GO terms that describe the molecular actions of gene products and their biological processes and cellular locations.^61^ The search terms, such as “differentiation,” “inflammatory,” “chromatin structure,” “cell cycle,” “cell death”, and “DNA damage” were used in this study to retrieve the lists of protein-producing genes involved in cellular differentiation, inflammatory processes, chromatin structure regulation, cell cycle, and cellular death and DNA damage processes, respectively. The results associated with the search terms were further filtered for the genes that are for *Homo sapiens* and are protein-coding, as non-coding genes often serve regulatory functions, and direct implications of homozygous variants harbored in the regulatory non-coding genes are complex to infer. We used “neuronal differentiation” and “neuronal inflammatory” as GO search terms for genes involved in neuronal differentiation and inflammatory processes in neurons, respectively, which were identified as subsets of cellular differentiation and inflammatory processes. For oncogenes and tumor suppressor genes, we used an open-source databases, oncogene database and TSGenes 2.0, which provide comprehensively curated human oncogenes^62^ and tumor suppressor genes (https://bioinfo.uth.edu/TSGene/). From the curated list, similar to the GO analysis, only the protein-coding oncogenes and tumor suppressor genes were used for analysis. Genomic regions associated with NDD phenotype and pathogenic CNVs were curated using DECIPHER (Database of Chromosomal Imbalance and Phenotype Using Ensembl Resources) and the UK Biobank, which were cumulatively considered as pathogenic CNVs in our analysis.^33,34^ A comprehensive list of genes associated with ASD and NDD phenotypes was prioritized and categorized into two groups, high-confidence and low-confidence candidate genes, by Leblond et al, and genes were retrieved from the database developed by the same group (https://genetrek.pasteur.fr/).^32^

### Genome-wide and context-specific analysis of homozygosity

We measured individual genome-wide homozygosity for variants as the number of all homozygous non-reference alleles divided by the total number of genomic loci genotyped. Similarly, individual genome-wide homozygosity of common variants was measured as the number of homozygous single nucleotide variants with minor allele frequency (MAF) ≥ 0.01 divided by the total number of genomic loci genotyped. For context-specific analysis, individual homozygosity rate was measured as the number of homozygous single nucleotide variants with MAF ≥ 0.01 divided by the total number of genomic loci genotyped within the defined genomic regions, per implicated gene groups or CNV regions. Relatedly, individual genome-wide homozygosity of rare and ultra-rare variants was measured as the number of homozygous single nucleotide variants with MAF < 0.01 and MAF < 0.0001, respectively, divided by the total number of genomic loci genotyped within the defined genomic regions, per implicated gene groups.

### Variant annotation and filtering

To annotate variants with allele frequencies from The Genome Aggregation Database (gnomAD), we used filtered-based annotation (-buildver hg19-protocol gnomad211_exome, gnomad211_genome) via the command-line tool ANNOVAR.^63^ The variants were first annotated with the v2.1.1 data set genome sequences, and any variants missing allele frequency information were then annotated with the v2.1.1 exome sequences data set. The variants that still lacked allele frequency information after annotating subsequently with genome and exome sequences were considered non-reported variants and annotated as 0 allele frequency. Variants with minor allele frequency (MAF) ≥0.01 were considered as common variants while those with MAF < 0.01 as rare variants. To annotate variants with variant consequences, transcript type, and variant location (exon vs. intron), we used the web interface Ensembl Variant Effect Predictor Assembly GRCh37.p13 (https://useast.ensembl.org/Tools/VEP/).

### Pathway enrichment analysis

For each gene set with significantly increased homozygosity, the variants only located within exons, and introns with implication in splicing were retained to be conservative in identifying the most likely genes affected by the homozygous common variants. Genes harboring at least one homozygous common variant passing filtering criteria were subjected to pathway analysis. We used Qiagen Ingenuity Pathway Analysis (IPA) to infer the canonical pathways likely to be impacted by the homozygous variants in the identified genes. Benjamini-Hochberg correction was applied to reduce the false discovery rate (FDR).

### Collapsing analysis of homozygous variants

Collapsing analysis of homozygous variants was performed to identify candidate modifier genes in our PHTS dataset. This analysis method was adapted from non-weighted rare-variant collapsing analysis to reflect genes harboring the qualifying homozygous variants in association with the NDD/ASD phenotype, hence, the modifier genes.^64^ For this analysis, secondary stringent variant filtration was conducted in addition to the primary filtration of variants for pathway analysis to extract the homozygous common variants predicted to be deleterious and/or damaging with high-confidence by SIFT and PolyPhen with thresholds of < 0.05 and > 0.9, respectively. These qualifying variants (QV) were then collapsed per gene-level with score 0 indicating absence of QV and score 1 reflecting at least one QV found in a gene in an individual. Fisher’s exact test was used to test for association of NDD/ASD against the non-NDD group and considered the genes with unadjusted *p* value < 0.05 as suggestive modifier genes.

### Statistical analysis

Statistical significance of the difference in homozygosity between the PHTS phenotype groups was determined by two-tailed unpaired Student’s t-test for normally distributed data and by two-tailed unpaired Mann-Whitney test for data with non-normal distribution using GraphPad Prism version 9.1.1. Distribution normality was determined by D’Agostino & Pearson test also using GraphPad Prism. The *p* values were adjusted using Benjamini-Hochberg correction method to control for false discovery rates in the homozygosity analysis of context-specific gene sets. To determine the relatively high burdens of homozygous common variants in the gene sets analyzed with the NDD/ASD phenotype, the NDD/ASD and the non-NDD dataset were combined, from which the mean and standard deviation were calculated. Using above-one standard deviation (1SD) as the threshold, a contingency table was created, and Fisher’s exact test was performed to calculate odds ratios and corresponding *p* values using R Studio version 4.2.1. As a proof of principle, logistic regression analysis was performed to demonstrate the feasibility of using homozygosity burdens as predictor variables to make models for NDD and ASD phenotypes in PHTS. First, backward stepwise regression method was used to select the predictor variables that produce the optimal model using stepAIC function from MASS package in R. This method starts with saturated model and removes a predictor in each iteration based on the Akaike information criterion (AIC). Then the AIC is compared across all steps, and the final model with the lowest AIC is selected. Upon optimal model selection, the sex of study participants was added to make the final predictive models. Subsequently, 80% and 20% of the data was split for training and testing, respectively, for internal validation of the predictive logistic regression models via 10-fold cross validation using Caret package in R. Then the receiver operating characteristic curve (ROC) analysis was performed using pROC package in R. For all analyses, *p* values < 0.05 were considered as statistically significant.

## Figures and Tables

**Table 1:** Basic demographic and *PTEN* genotype data of study participants

	NDD^a^	ASD^b^	CANCER	OTHER
**Age at consent, years**				
Median	8	7	50	34
Range	1–58	2–49	3–85	3–72
**Sex, No. (%)**				
Male	81 (69)	42 (74)	32 (18)	45 (54)
Female	36 (31)	15 (26)	143 (82)	39 (46)
**PTEN variant types**				
Promoter	-	-	20	5
Missense	53	33	51	28
Nonsense	27	10	42	18
Alternative splicing	10	4	16	8
Frameshift truncating	20	8	32	16
In-frame insertion/deletion	2	-	2	-
Exon deletion/duplication	3	2	11	8
Whole gene deletion	2	-	1	1
**PTEN variant classification**				
Pathogenic/likely pathogenic	108	51	131	73
Benign/likely benign	-	-	11	1
Unknown significance	9	6	33	10

a NDD has 14 patients comorbid with cancer, three of whom are with ASD

b ASD is a subset of NDD group.

**Table 2:** Summary of suggestive modifier genes associated with NDD phenotype in PHTS

Gene	No. observed in NDD (%)	OR (90% CI)	Unadjusted *P*	Directionality of association to NDD	Pathways involved
**OR51S1**	35 (61.4%)	1.97 (1.25–3.12)	0.010	Positive	-
**CPA6**	5 (8.8%)	11.44 (1.62–270.5)	0.012	Positive	-
**KBTBD13**	12 (21.1%)	0.46 (0.24–0.83)	0.018	Negative	-
**INADL**	6 (10.5%)	4.59 (1.19–21.54)	0.029	Positive	-
**HNF1A**	19 (33.3%)	2.19 (1.19–4.04)	0.029	Positive	Hepatic Fibrosis Signaling Pathway, Osteoarthritis Pathway, Hepatic Cholestasis, and S100 Family Signaling Pathway
**USP31**	19 (33.3%)	2.19 (1.19–4.04)	0.029	Positive	-
**GABRA4**	22 (38.6%)	2.07 (1.17–3.64)	0.029	Positive	Neuroinflammation Signaling Pathway; Neurovascular Coupling Signaling Pathway; GABA Receptor Signaling
**OPRM1**	3 (5.3%)	Inf (1.30-Inf)	0.030	Positive	Phagosome Formation; CREB Signaling in Neurons; cAMP-mediated signaling; S100 Family Signaling Pathway
**LRP8**	11 (19.3%)	0.47 (0.24–0.87)	0.031	Negative	Synaptogenesis Signaling Pathway; Pyroptosis Signaling Pathway; Oxytocin in Brain Signaling Pathway; Reelin Signaling in Neurons
**BPIFB6**	4 (7.0%)	9.08 (1.17–222.98)	0.034	Positive	-
**LAMA5**	4 (7.0%)	9.08 (1.17–222.98)	0.034	Positive	GP6 Signaling; Myelination Signaling Pathway; Would Healing Signaling Pathway; and CDK5 Signaling
**TLR3**	3 (5.3%)	0.28 (0.07–0.84)	0.042	Negative	Phagosome Formation; Pathogen Induced Cytokine Strom Signaling; Neuroinflammation Signaling Pathway; LXR/RXR Activation

**Table 3:** Summary of suggestive modifier genes associated with ASD phenotype in PHTS

Gene	No. observed in ASD (%)	OR (90% CI)	Unadjusted *P*	Directionality of association to ASD	Pathways(s) involved	SFARI gene (score)^a^
**USP31**	12 (21.1%)	3.01 (1.43–6.16)	0.007	Positive	-	No, but other genes in the same family found, such as USP15 (2), UPS30 (3), USP45 (2), USP7 (2, S), USP9X (1,S), and USP9Y (2)
**FRAS1**	2 (3.5%)	0.19 (0.03–0.65)	0.010	Negative	-	No
**LRP8**	3 (5.3%)	0.25 (0.06–0.72)	0.015	Negative	Synaptogenesis Signaling Pathway; Reelin Signaling in Neurons	No, but other genes in the same family found, such as LRP1 (2) and LRP2 (2)
**CX3CR1**	6 (10.5%)	4.21 (1.36–12.70)	0.016	Positive	Neuroinflammation Signaling Pathway; S100 Family Signaling Pathway; Phagosome Formation; CREB Signaling in Neurons	Yes (2)
**SPATA16**	4 (7.0%)	6.38 (1.35–33.27)	0.022	Positive	-	No
**OR51S1**	18 (31.6%)	2.13 (1.17–3.82)	0.028	Positive	-	No, but other genes in the same family found, such as OR1C1 (2), OR2M4 (2), OR2T10 (2), and OR52M1 (2)
**SPTBN5**	11 (19.3%)	2.57 (1.20–5.30)	0.028	Positive	-	No, but other genes in the same family found, such as SPTBN1 (2, S)
**BST1**	2 (3.5%)	Inf (1.32-Inf)	0.032	Positive	NAD Signaling Pathway	Yes (2)
**SCML4**	2 (3.5%)	Inf (1.32-Inf)	0.032	Positive	-	No
**OPRM1**	2 (3.5%)	Inf (1.32-Inf)	0.032	Positive	Phagosome Formation, CREB Signaling in Neurons; S100 Family Signaling Pathway	No
**DLG1**	4 (7.0%)	4.78 (1.10–20.60)	0.038	Positive	Cardiac Hypertorphy Signaling; Sertoli Cell-Sertoli Cell Junction Signaling	Yes (2)

a For SFARI genes, scores of 1, 2, and S denote high confidence, strong candidate, and syndromic genes, respectively, for ASD.

## Data Availability

The Cleveland Clinic Foundation institutional IRB and Legal Department do not permit clinical information or original genomics data reposited in a publicly accessible database at this time (by policy). Requests for such data relevant to this paper should be made to the corresponding author C.E. (engc@ccf.org). Thereafter, the Legal Department will ask for material transfer and data sharing agreements to be executed. Analyzed genomic data related to this study are included in the figures, table, and Supplementary Data 1–4.
